# Lipid Profile Abnormalities in Newly Diagnosed Primary Hypothyroidism in a Tertiary Care Centre of Western Nepal: A Descriptive Cross-sectional Study

**DOI:** 10.31729/jnma.6809

**Published:** 2021-08-31

**Authors:** Prabin Khatri, Aryan Neupane, Ashish Banjade, Suman Sapkota, Smile Kharel, Ashmita Chhetri, Dipesh Sharma, Saphal Nath Subedi, Pradip Chhetri

**Affiliations:** 1 Department of Internal Medicine, Universal College of Medical Sciences, Bhairahawa, Nepal; 2 Universal College of Medical Sciences, Bhairahawa, Nepal; 3 Karuna Hospital, Kathmandu, Nepal; 4 Department of Community Medicine, Universal College of Medical Sciences, Bhairahawa, Nepal

**Keywords:** *dyslipidemia*, *hypothyroidism*, *lipids*

## Abstract

**Introduction:**

Thyroid hormones have a major influence on synthesis, mobilization and metabolism of lipids. Hypothyroidism accounts for a notable cause of secondary dyslipidemia. This can increase the risk for cardiovascular morbidity and mortality. This study was performed to find out the prevalence of lipid profile abnormalities in newly diagnosed primary hypothyroid states.

**Methods:**

This descriptive cross-sectional study was conducted among 71 patients in the context of newly diagnosed primary hypothyroidism patients visiting outpatient department of internal medicine from 9th December 2018 to 30th June 2020 after taking ethical clearance from Institutional Review Committee. Case screening for lipid profile changes was performed at the time of diagnosis of primary hypothyroidism. A convenience sampling method was used. Data entry and descriptive analysis were done in Statistical Package for the Social Sciences version 16.0. Point estimate at 95% Confidence Interval was calculated along with frequency and proportion for binary data.

**Results:**

In this study including 71 cases of newly diagnosed primary hypothyroidism, 49 (69.0%) (95% Confidence Interval = 58.24 - 79.76) had abnormal lipid profiles. Among them, 5 (38.5%) out of 13 (18.3%) cases of subclinical hypothyroidism and 44 (75.9%) out of 58 (81.7%) cases of overt hypothyroidism had abnormal lipid profiles.

**Conclusions:**

The prevalence of abnormal lipid profile parameters was similar to the study done in various studies in similar settings except for high-density lipid which showed both similarity and dissimilarity with other studies. Our study suggested that all newly diagnosed cases of primary hypothyroidism are to be investigated for dyslipidemia thus ensuring early treatment and prevention of complications.

## INTRODUCTION

Hypothyroidism is a clinical syndrome that results in deficiency of thyroid hormone. When the defect is within the thyroid gland it is called primary hypothyroidism and when indirect pathologies contribute to the decrease in hormone levels it is secondary hypothyroidism.^1^ Thyroid hormones have a major influence on synthesis, mobilization and metabolism of lipids.^2^ With decline in thyroid function, total cholesterol and low-density lipoprotein cholesterol tend to increase.^3^ Alteration in lipid profile in hypothyroid states is largely due to a decrease in low-density lipoprotein (LDL) receptor activity.^4^ Thus hypothyroidism accounts for a notable cause of secondary dyslipidemia.^5^

In Nepal, about 4.32% of the general public have thyroid disorders.^6^ Dyslipidemia can increase the risk for cardiovascular disease and, probably, mortality so lipid profile must be regularly monitored in hypothyroid patients.

This study was performed to find out the prevalence of lipid profile abnormalities in newly diagnosed primary hypothyroid states.

## METHODS

This study, a descriptive cross-sectional study, was conducted in Universal College of Medical Sciences Teaching Hospital, Bhairahawa, Nepal in the context of newly diagnosed primary hypothyroidism patients visiting the outpatient department of internal medicine. Case screening for lipid profile changes was performed at the time of diagnosis of primary hypothyroidism. The study was conducted for one and half year's duration from 9th December 2018 to 30th June 2020. The permission to conduct this research was taken from the institutional review committee of UCMS-TH (UCMS/IRC/212/18) on 8^th^ December 2018. Written consent was obtained from all the participants. Convenience sampling method was used and the sample size was calculated by using the formula,

n = Z^2^ × p × q / e^2^

  = (1.96)^2^ × 0.0885 × 0.9115 / (0.07)^2^

  = 64

Where,

n = sample size
Z = 1.96 at 95% Confidence Interval (CI)
p = prevalence of Primary Hypothyroidism, 8.85%^7^
q = 1 - p = 0.9115
e = allowable error, 7%

Then, taking 10% non-respondent rate the total sample size was 71.

All newly diagnosed cases of primary hypothyroidism presenting to the OPD of Internal Medicine in Universal College of Medical Sciences& Teaching Hospital (UCMSTH) were taken as the study population. Patients below 18 years of age, those with secondary hypothyroidism, those with underlying heart disease, chronic kidney disease, liver disease, diabetes mellitus or any other endocrinal disorder, under medications that alter the thyroid function (example: Betablockers, lithium, oral contraceptive pills, steroids, and amiodarone), patients who consume alcohol, patients taking lipid-lowering medications, those refusing participation in the study, and pregnant patients were excluded from the study.

Thyroid function test measured using in vitro chemiluminescent immunoassay for the quantitative determination of free T3 (fT3), T4 and TSH in human serum using the MAGLUMI series fully auto chemiluminescence immunoassay analyzer. Thyroid hormone abnormalities were made if patients thyroid hormones were outside the normal values; fT3 (2.0-4.2 pg/ml), fT4 (8.9-17.2 pg/ml) and TSH (0.3-4.5 mIU/ml).

The assay for (High-density lipoprotein) HDL Estimation combines two specific steps: in the first step chylomicrons, (Very low-density lipoprotein) VLDL and (Low-density lipoprotein) LDL cholesterol are specifically eliminated and destroyed by enzymatic reactions. In the second step, the remaining cholesterol from the HDL fraction is determined by well-established specific enzymatic reactions in the presence of specific surfactants for HDL.

The data were collected and filled up in proforma for each patient by the investigator. The data collected were entered in Statistical Package for Social Sciences (SPSS) Version 16. Point estimate at 95% Confidence Interval was calculated along with frequency and proportion for binary data.

## RESULTS

Out of the total 71 newly diagnosed primary hypothyroid patients, 49 (69.0%) (95% CI = 58.24-79.76) had abnormal lipid profiles. Among them, 5 (38.5%) out of 13 cases of subclinical hypothyroidism and 44 (75.9%) out of 58 cases of overt hypothyroidism had abnormal lipid profiles.

**Table 1:** Distribution of lipid abnormality among different types of primary hypothyroidism.

Lipid Abnormalities	Abnormal n (%)	Normal n (%)	Total n (%)
Subclinical	5 (38.5)	8(61.5)	13 (18.3)
Overt	44 (75.9)	14 (24.1)	58 (81.7)
Total	49 (69.0)	22 (31.0)	71 (100)

Table 2 shows the distribution of various components of lipid profile with types of primary hypothyroidism (Table 2).

**Table 2:** Distribution of various components of lipid profile with types of primary hypothyroidism.

Lipid Profile		Sub clinical n (%)	Overt n (%)	Total n (%)
Total Cholesterol	Normal	8(61.5)	38 (65.5)	46 (64.7)
	High	5 (38.5)	20 (34.5)	25 (35.3)
LDL	Normal	8(61.5)	25 (43.1)	33 (46.4)
	High	5 (38.5)	33 (56.9)	38 (53.6)
HDL	Normal	10 (76.5)	22 (37.9)	32 (45.0)
	Low	3 (23.1)	36 (62.1)	39 (55.0)
Triglyceride	Normal	9 (69.2)	18 (31)	27 (38.0)
	High	4 (30.8)	40 (69)	44 (62.0)

Table 3 shows the distribution of study patients with respect to thyroid function tests (Table 3).

**Table 3:** Distribution of study patients according to thyroid function test.

Thyroid function test		n (%)	Mean±S.D.
T3	Subclinical	13 (18.3)	2.37±1.05
	Overt	58 (81.7)	1.76±1.40
T4	Subclinical	13 (18.3)	9.56±1.56
	Overt	58 (81.7)	4.27±2.10
TSH	Subclinical	13 (18.3)	8.73±0.65
	Overt	58 (81.7)	41.72±31.89

Regarding presenting symptom, as shown in Table 4, constipation 36 (50.70%) was the most common followed by weight gain 30 (42.30%). Other symptoms were lethargy 28 (39.40%), dyspnea 17 (23.90%), cold intolerance 17 (23.90%), menstrual irregularities 16 (22.50%), and dry skin 14 (19.70%) (Table 4).

**Table 4:** Distribution of study patients according to clinical presentation.

Presenting Complaints	n (%)
Constipation	36 (50.7)
Weight Gain	30 (42.3)
Lethargy	28 (39.4)
Dyspnea	17 (23.9)
Cold Intolerance	17 (23.9)
Menstrual Irregularities	16 (22.5)
Dry Skin	14 (19.7)

**Figure 1:**
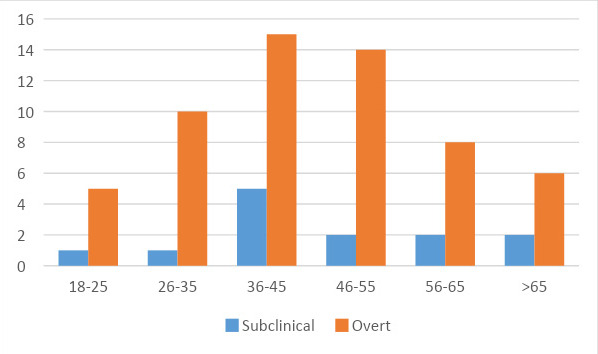
Distribution of study patients according to age group.

Out of total population of study, 52 (73.2%) were female and 19 (26.8%) were male. Figure 1 shows distribution of study patients according to age group, most of them 15 (21.13%) were in age group 36-45 years.

## DISCUSSION

In this study, Lipid profile abnormalities were studied in newly diagnosed primary hypothyroid patients attending the outpatient department at our centre. A total of 71 cases were enrolled for the study including subclinical and overt hypothyroidism.

In this study among 71 cases, 19 (26.8%) were males and 52 (73.3%) females. A study was done by Mahato RV, et al. also had similar sex distribution.^8^ Higher prevalence in females can be related to female sex hormones.^9^ The most common age group was 36-45 years, which is 28.2% of total cases. Dangi V and Meena RS did a study where most cases were of age group 31-40 years.^10^ In our study, 13 cases (18.3%) were of subclinical hypothyroidism whereas, 58 cases (81.7%) were of overt hypothyroidism which is different from a study by Aryal M, et al. which demonstrated an equal number of subclinical and overt hypothyroidism.^11^

In a study by Kumbhalkar, et al. the most common symptom in hypothyroidism was weight gain followed by easy fatigability, dryness of skin, menstrual abnormalities, constipation, depression, change in voice.^12^ Compared to this the most common presenting complaint in our study was constipation followed by weight gain. A decrease in gastrointestinal motility is responsible for constipation.^13^ In hypothyroid states, there is decreased basal metabolism and thermogenesis, an accumulation of hyaluronic acid and a decreased renal flow. These factors collectively lead to water retention and thus weight gain.^14^ Among others symptoms were lethargy, dyspnea, menstrual irregularities, and dry skin.

In this study including 71 cases, 49 (69.0%) had abnormal lipid profiles. Among them, 5 (10.2%) were of subclinical hypothyroidism and 44 (89.8%) were of overt hypothyroidism. A study by Khan, et al., found abnormalities in total cholesterol, LDL, HDL, and TG in hypothyroid patients.^15^ In another study done in Pakistan by Malik A, et al., significant changes in total cholesterol, HDL, Triglyceride and LDL compared to control were found.^16^

Similar changes in lipid profile were observed in our study too. Our study showed an increase in total cholesterol, LDL and TG whereas a decrease in HDL. A study was done in Dhaka by Khan, et al., showed a decrease in HDL levels in cases of hypothyroidism which is relatable to our study. Whereas a study done by Olukoga^17^ showed an increase in levels of HDL in hypothyroidism which is in contrast to our study. So our study was relatable to some studies and in contrast to some studies. A larger sample with case and controls and multicenter studies are needed to address these issues.

The reason behind the increase in LDL levels is due to decreased LDL receptor activity which leads to reduced catabolism of LDL.^18^ Increased activity of CETP (Cholesteryl ester transport protein) and hepatic lipase in hypothyroid patients is responsible for the decrease in HDL levels. Similarly decreased stimulation of lipoprotein lipase in hypothyroid patients leads to increased levels of TG due to decreased catabolism of TG-rich lipoproteins. Thyroid hormone is responsible for the up-regulation of apolipoprotein AV (ApoAV). ApoAV plays an important role in TG regulation. Decreased activity of ApoAV in hypothyroidism may also explain levels of TGs.^19^

Since the study is conducted on a single institution the findings can't be used to represent a general population. So other large scale multi-institutional studies with bigger sample size is needed in the future.

## CONCLUSIONS

Dyslipidemia was frequently seen in patients with newly diagnosed primary hypothyroidism. Our study suggested that all newly diagnosed cases of primary hypothyroidism are to be investigated for dyslipidemia thus ensuring early treatment and prevention of complications. Screening for thyroid dysfunction is needed in all patients with dyslipidemia. Also, patients with hypothyroidism should make changes to lifestyle and diet to prevent further cardiovascular morbidities and mortalities.
